# The Role of IRE1α in the Degradation of Insulin mRNA in Pancreatic β-Cells

**DOI:** 10.1371/journal.pone.0001648

**Published:** 2008-02-20

**Authors:** Kathryn L. Lipson, Rajarshi Ghosh, Fumihiko Urano

**Affiliations:** 1 Program in Gene Function and Expression, University of Massachusetts Medical School, Worcester, Massachusetts, United States of America; 2 Program in Molecular Medicine, University of Massachusetts Medical School, Worcester, Massachusetts, United States of America; University of California Los Angeles, United States of America

## Abstract

**Background:**

The endoplasmic reticulum (ER) is a cellular compartment for the biosynthesis and folding of newly synthesized secretory proteins such as insulin. Perturbations to ER homeostasis cause ER stress and subsequently activate cell signaling pathways, collectively known as the Unfolded Protein Response (UPR). IRE1α is a central component of the UPR. In pancreatic β-cells, IRE1α also functions in the regulation of insulin biosynthesis.

**Principal Findings:**

Here we report that hyperactivation of IRE1α caused by chronic high glucose treatment or IRE1α overexpression leads to insulin mRNA degradation in pancreatic β-cells. Inhibition of IRE1α signaling using its dominant negative form prevents insulin mRNA degradation. Islets from mice heterozygous for IRE1α retain expression of more insulin mRNA after chronic high glucose treatment than do their wild-type littermates.

**Conclusions/Significance:**

These results reveal a role of IRE1α in insulin mRNA expression under ER stress conditions caused by chronic high glucose. The rapid degradation of insulin mRNA could provide immediate relief for the ER and free up the translocation machinery. Thus, this mechanism would preserve ER homeostasis and help ensure that the insulin already inside the ER can be properly folded and secreted. This adaptation may be crucial for the maintenance of β-cell homeostasis and may explain why the β-cells of type 2 diabetic patients with chronic hyperglycemia stop producing insulin in the absence of apoptosis. This mechanism may also be involved in suppression of the autoimmune type 1 diabetes by reducing the amount of misfolded insulin, which could be a source of “neo-autoantigens.”

## Introduction

The endoplasmic reticulum (ER) is a cellular compartment for the biosynthesis and folding of newly synthesized secretory proteins such as insulin. Perturbations in ER function cause dysregulation of ER homeostasis, leading to ER stress [1], [2]. Cells cope with ER stress by activating an ER stress signaling cascade called the unfolded protein response (UPR). This activation results in the upregulation of gene expression for molecular chaperones, expands the size of the ER, decreases general protein translation to reduce the ER workload, and degrades abnormal proteins accumulated in the ER [3], [4].

As long as ER stress signaling via the UPR can keep ER stress levels under control, cells can perform their normal functions. However, under some pathological conditions, such as obesity and progressive neurodegeneration, a high level of chronic ER stress persists, leading to cell dysfunction and death [5], [6], [7]. It has been suggested that chronic and high levels of ER stress have a function in β-cell dysfunction and glucose toxicity. In glucose toxicity, insulin secretion by β-cells is impaired in response to stimulation by glucose; the condition is characterized by a sharp decline in insulin gene expression [8], [9]. Numerous studies have shown that impaired β-cell dysfunction can be improved by treatment of the hyperglycemia [8], [9], suggesting that identifying the molecular mechanisms involved in β-cell glucose toxicity may provide new therapeutic targets for diabetes.

Inositol requiring 1 α (IRE1α) is a transmembrane protein kinase/endoribonuclease that is localized in the ER and activated by ER stress. Unfolded proteins in the ER are sensed by the IRE1-BiP complex, which causes dimerization, autophosphorylation, and subsequent activation of IRE1. Activated IRE1 splices X-box binding protein-1 (Xbp-1) mRNA in the cytoplasm, leading to synthesis of the active transcription factor Xbp-1 and upregulation of ER stress-response genes [10], [11]. In addition, IRE1 can promote cleavage of the 28S ribosomal RNA [12], as well as mRNA encoding IRE1 [13].

In metazoans, IRE1 activation initiates two separate signaling cascades: an XBP-1- dependent pathway that upregulates ER stress response genes and an XBP-1-independent pathway involving specific cleavage and subsequent degradation of sets of translating mRNAs on the ER membrane [14]. This response complements other components of the UPR, selectively halting protein synthesis and clearing the translocation machinery when translating mRNAs are overloading the ER and causing ER stress. It has been suggested that this specific mRNA degradation may result from IRE1 focusing on messages that present the most immediate challenge to the translocation and folding machinery [14].

In mice and rats, the two insulin gene transcripts are the most abundantly transcribed mRNAs in β-cells bound for translation through the ER membrane [15]. Global profiling of genes modified by ER stress in β-cells has shown that upon induction of ER stress, Insulin 1 and Insulin 2 mRNA are quickly degraded, leading to significantly decreased levels of these mRNAs Pirot et al. (2007). We have shown that hyperactivation of IRE1 is correlated with reduction in insulin mRNA expression in pancreatic β-cells (Lipson et al., 2006). Based on these observations, we hypothesized that when ER folding capacity is overwhelmed, IRE1 initiates endonucleolytic cleavage of mRNA encoding insulin, the major secretory protein in pancreatic β-cells. Here we report that IRE1α hyperactivation has a function in insulin mRNA reduction under chronic high-glucose conditions and implicate ER stress in the molecular mechanisms of glucose toxicity.

## Results

### Chronic high glucose causes ER stress and reduces insulin gene expression in pancreatic β-cells

To investigate the effect of chronic high-glucose treatment on expression levels of insulin and ER stress response genes, we treated INS-1 832/13 cells and primary mouse islets with increasing concentrations of glucose for 24 and 72 hr, then measured expression levels of Insulin 1 and Insulin 2 gene expression, as well as expression of several well-known markers of ER stress.

**Figure 1:**
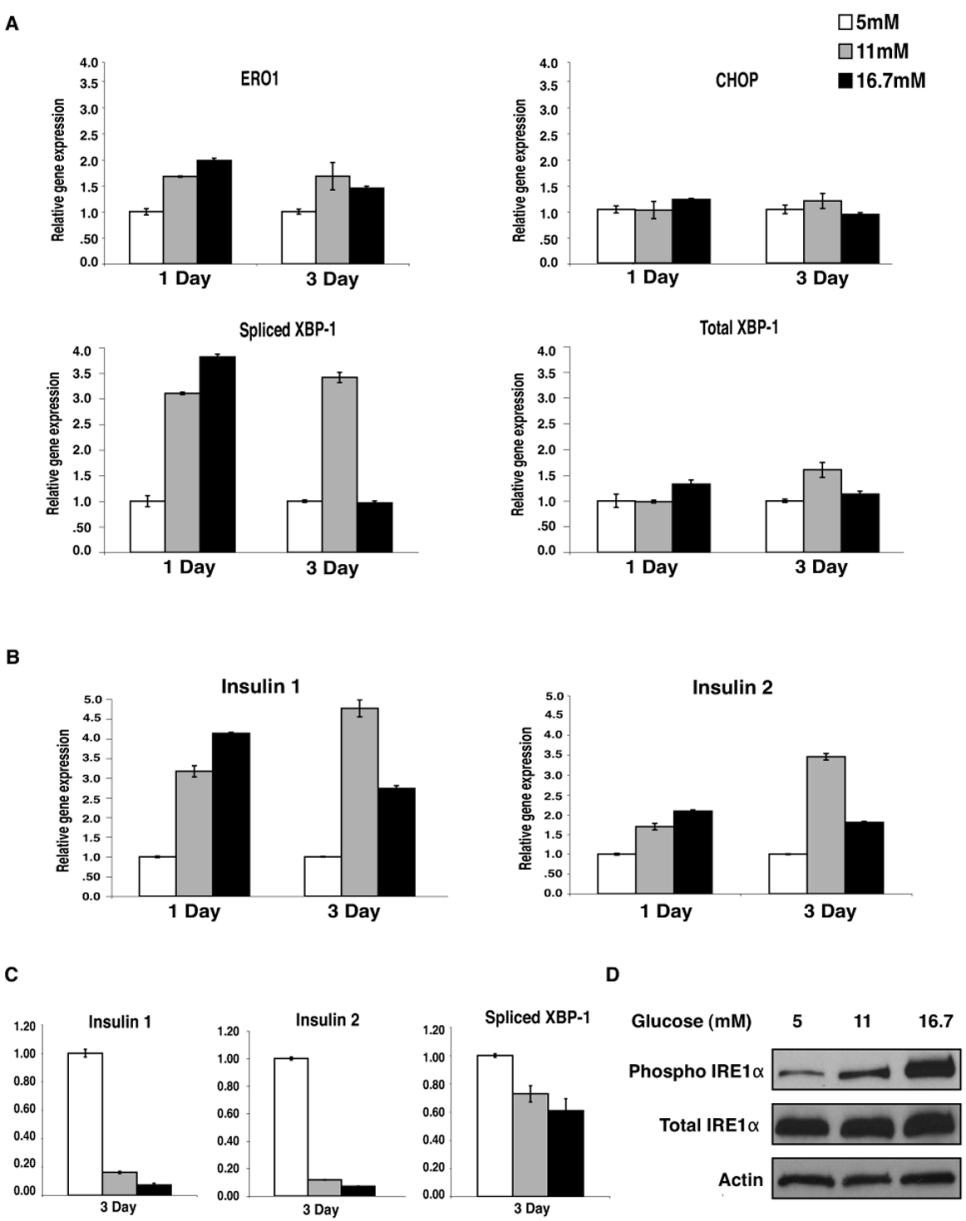
Chronic high-glucose treatment causes ER stress in islets and insulinoma cells, resulting in a reduction in insulin gene expression. (A) Islets pooled from 6 mice were treated with 5 mM, 11 mM, or 16.7 mM glucose for 24 or 72 hr. Expression levels of Ero1α, Chop, spliced Xbp-1, and total Xbp-1 were measured by real- time PCR (n = 2). (B) Islets pooled from 6 mice were treated with 5 mM, 11 mM, or 16.7 mM glucose for 24 or 72 hr. Expression levels of Insulin 1 and Insulin 2 were measured by real time PCR (n = 2). (C) INS-1 832/13 cells were pretreated for 12 hr with 5 mM glucose, then treated with 5 mM, 11 mM, or 16.7 mM glucose for 72 hr. Expression levels of Insulin 1, Insulin 2, and spliced Xbp-1 were measured by real time PCR (n = 3; values are mean±SEM). (D) INS-1 832/13 cells were pretreated for 12 hr with 5 mM glucose, then treated with 5 mM, 11 mM, or 16.7 mM glucose for 72 hr. Total IRE1α, phosphorylated IRE1α, and actin were measured by immunoblot.

After 24 hr, both Insulin 1 and Insulin 2 gene expression increased with increasing glucose. Expression levels of spliced Xbp-1 and Ero1α, ER stress markers, also increased with glucose concentration. The expression of Chop, an ER stress marker of apoptosis remained the same, suggesting that chronic glucose treatment causes mild ER stress but not cell death (Fig. 1A). Seventy-two hr treatment with high glucose caused a dramatic reduction in both Insulin 1 and Insulin 2 gene expression, an indication of glucose toxicity (Fig. 1B). We also observed a decrease in Xbp-1 splicing and other ER stress markers in the islets after 72 hr treatment with 16.7 mM glucose (Fig. 1A), and with both 11 mM and 16.7 mM in INS-1 832/13 cells (Fig. 1C). This decrease in Xbp-1 splicing directly correlated with the decrease in Insulin 1 and Insulin 2 gene expression. However, we observed that phosphorylation of Ire1α increased as glucose concentration increased, and was much stronger after 72 hr of treatment despite the reduction in XBP-1 splicing (Fig. 1D).

### IRE1α overexpression correlates with reduced insulin mRNA

**Figure 2:**
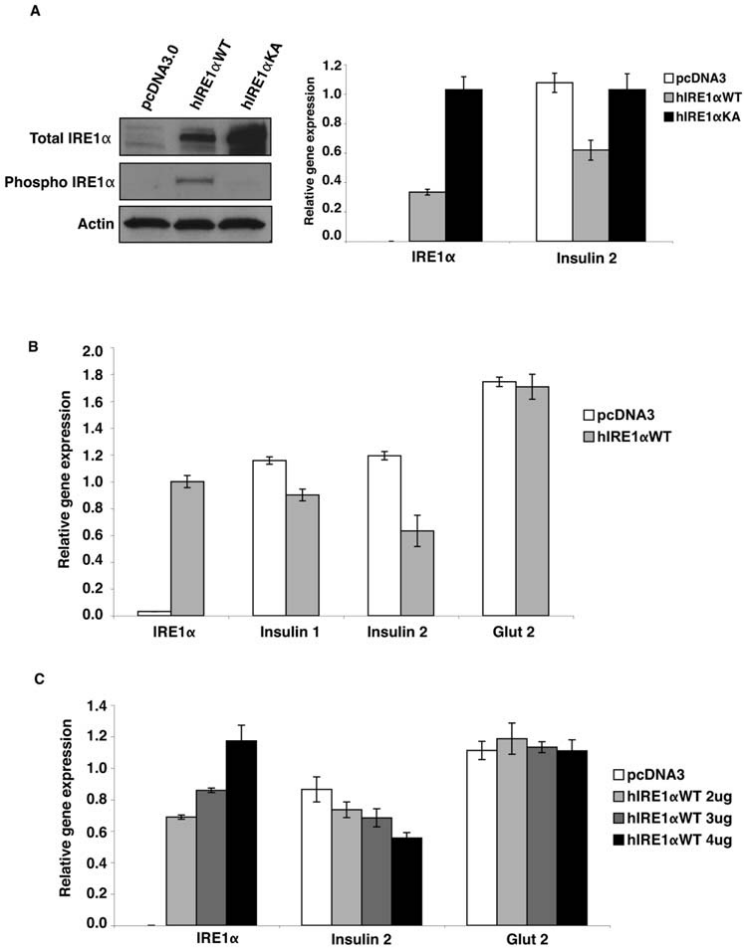
Overexpression of IRE1α correlates with reduced Insulin mRNA in cultured cells. (A) COS-7 cells were transfected with mouse Insulin 2 and cultured for 24 hr. Cells were then split onto 3 plates and transfected again with wild-type human Ire1α; IRE1α WT, a kinase/endoribonuclease inactive mutant human Ire1α; IRE1α KA; or pcDNA3 control. They were then cultured for 24 hr. Protein and RNA were collected from the same plates. Total IRE1α, phosphorylated IRE1α, and actin were measured by immunoblot. Expression levels of human IRE1α and mouse Insulin 2 were measured by real time PCR (n = 3; values are mean±SEM). (B) INS-1 832/13 cells were transfected with human IRE1α WT or pcDNA3 control and cultured for 24 hr. Expression levels of human IRE1α, endogenous rat insulin 1, insulin 2, and glucose transporter 2 (glut 2) were measured by real time PCR (n = 3; values are mean±SEM). (C) INS-1 832/13 cells were transfected with either pcDNA3 control or increasing concentrations of human IRE1α WT and cultured for 24 hr. Expression levels of human IRE1α, endogenous rat insulin 1, insulin 2, and glucose transporter 2 (glut 2) were measured by real-time PCR (n = 3; values are mean±SEM).

Our results suggested that chronic high glucose activates an Xbp-1 independent Ire1α signaling cascade. The strong activation of Ire1α, combined with the loss of Xbp-1 splicing during chronic high-glucose treatment, led us to hypothesize that IRE1α itself may have a direct function in the degradation of insulin mRNA. To test this hypothesis, we transfected COS-7 cells with mouse Insulin 2 expression plasmid, then transfected wild-type human IRE1α expression plasmid or kinase/endoribonuclease inactive dominant-negative mutant K599A IRE1α expression plasmid in these cells, then measured insulin gene expression (Fig. 2A).

We observed a reduction in Insulin 2 mRNA in cells overexpressing wild-type IRE1α, but not in cells overexpressing mutant K599A IRE1α or the pcDNA3 control.

We also transfected either wild-type human IRE1α or K599A IRE1α expression plasmid in INS-1 832/13 cells and measured endogenous Insulin 1 and Insulin 2 mRNA expression (Fig. 2B). We found downregulation of both Insulin 1 and Insulin 2 gene expression only in cells expressing wild-type IRE1α.

To study the correlation between IRE1α expression levels and insulin gene expression, we expressed increasing amounts of wild-type IRE1α in INS-1 832/13 cells and measured expression levels of insulin. The results indicated a dose-dependent response to the amount of IRE1α expressed and the fold reduction in endogenous insulin mRNA (Fig. 2C). Taken together, these data show a firm correlation between strong activation of IRE1α and the degradation of insulin mRNA independent of glucose concentration.

### Inhibition of IRE1α activation by a dominant negative mutant blocks both high glucose induced and thapsigargin induced insulin mRNA degradation

**Figure 3:**
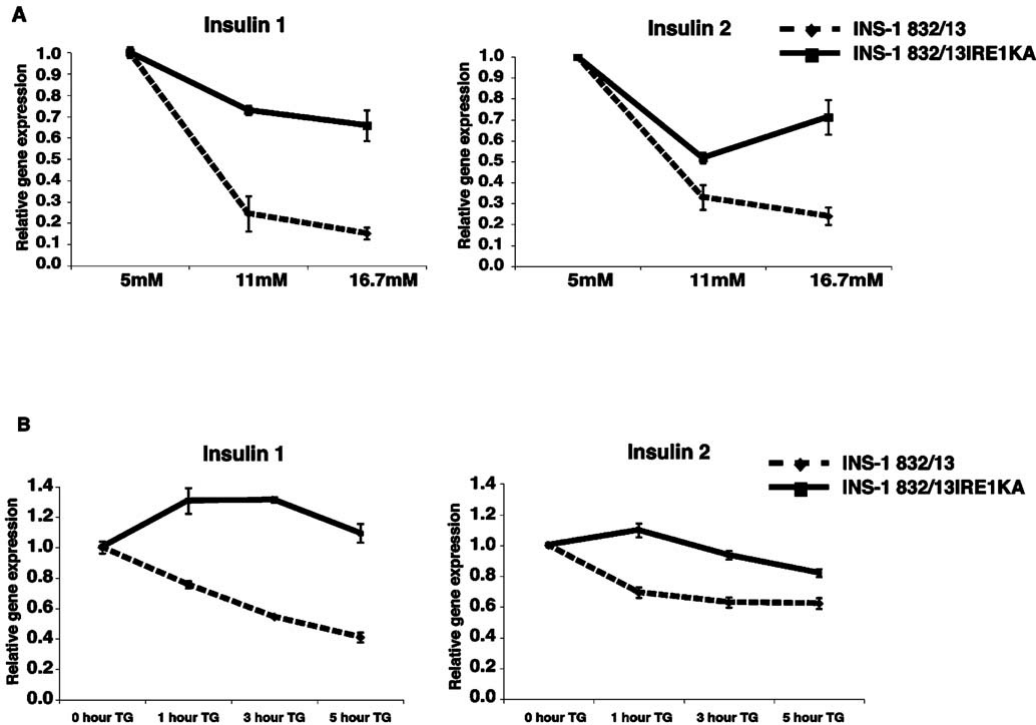
Cells expressing mutant IRE1α resist both chemical- and glucose-induced Insulin mRNA degradation. (A) pTetRINS-1 832/13 cells (control) and pTetRINS-1832/13IRE1αKA cells (stably expressing tetracycline-responsive K599A mutant IRE1α) were treated with doxycycline for 24 hr with 5 mM glucose to induce mutant IRE1α. Cells were then treated with 5 mM, 11 mM, or 16.7 mM glucose with doxycycline for 72 hr. Degradation of mRNA was assessed by measuring the expression of Insulin 1 and Insulin 2 by real-time PCR (n = 3; values are mean±SEM). (B) pTetRINS-1 832/13 cells (control) and pTetRINS-1832/13IRE1αKA cells (stably expressing tetracycline-responsive K599A mutant IRE1α) were treated with doxycycline for 24 hr to induce mutant IRE1α. mRNA transcription was attenuated by treating cells with 100 µg/mL actinomycin D for 1 hr. To induce degradation of insulin mRNA, 1 µM thapsigargin was added to the medium for 0, 1, 3 and 5 hr. mRNA degradation was assessed by measuring expression of Insulin 1 and Insulin 2 by real-time PCR (n = 3; values are mean±SEM).

To test the hypothesis that IRE1α is directly involved in the reduction of insulin mRNA on exposure to chronic high glucose, we generated INS-1 832/13 cell lines expressing the dominant-negative mutant K599A IRE1α, using a lentivirus-based doxycycline-mediated induction system. We induced expression of K599A IRE1α or empty vector control in INS-1 832/13 cells, then challenged them with increasing concentrations of glucose for 72 hr (Fig. 3A). As compared to the INS-1 832/13 control cells, cells stably expressing the dominant-negative mutant K599A IRE1α resisted the high glucose-induced reduction in insulin mRNA and had higher gene expression for both Insulin 1 and Insulin 2.

Thapsigargin, a chemical ER stress inducer, causes a significant decrease in the transcript levels of Insulin 1 and Insulin 2 [16]. To determine whether IRE1α is responsible for this chemically induced ER stress decrease in insulin mRNA expression, we used actinomycin D to attenuate mRNA transcription, then challenged the cells with thapsigargin to induce Insulin 1 and Insulin 2 mRNA degradation. Cells expressing the dominant-negative mutant K599A IRE1α resisted the thapsigargin-induced decrease in both Insulin 1 and Insulin 2 gene transcripts observed in control cells (Fig. 3B).

### Islets from mice heterozygous for IRE1α are resistant to high-glucose-mediated reduction in insulin mRNA expression

**Figure 4:**
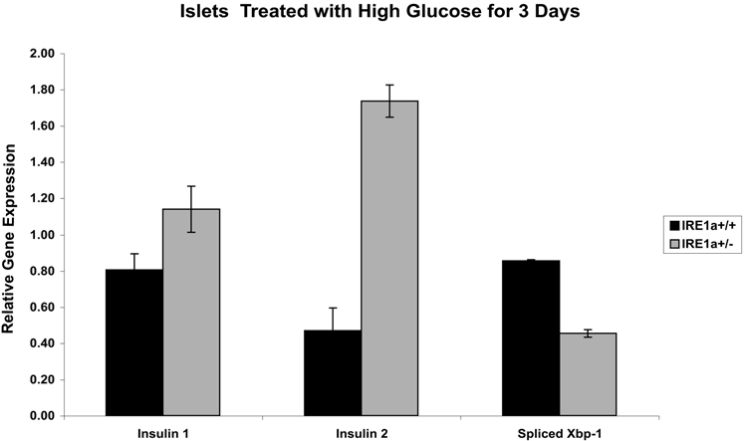
IRE1α heterozygous islets resist the negative effects of chronic high glucose. IRE1α WT or IRE1α heterozygous mouse islets were treated with 16.7 mM glucose for 72 hr. Expression of Insulin 1, Insulin 2, and spliced XBP-1 were measured by quantitative real-time PCR.

We observed that cells in which strong activation of IRE1α was partially blocked by use of a mutant form of IRE1α were more resistant than control cells to the glucotoxic effects of chronic high glucose on insulin gene expression. We then tested whether islets from mice heterozygous for IRE1α are also more resistant to chronic high glucose. We treated islets from mice that were heterozygous for IRE1α or islets from their wild-type littermates with 16.7 mM glucose for 72 hr, then measured Insulin 1 and Insulin 2 gene expression and Xbp-1 splicing (Fig. 4). Heterozygous mouse islets were more resistant to the glucotoxic effects of chronic high glucose exposure in that they had higher Insulin 1 and Insulin 2 gene expression than did their wild-type littermate controls. However, splicing of Xbp-1 was lower in the IRE1α heterozygotes, suggesting that these mice were also more resistant to ER stress induced by chronic high glucose. These results suggest that IRE1α acts directly in the reduction of insulin mRNA under chronic ER stress conditions and that blocking the activation of IRE1α under these conditions can protect cells from negative effects.

## Discussion

Our results demonstrate a genetic and biochemical linkage between ER stress signaling and reduction in insulin mRNA expression in pancreatic β-cells under chronic high glucose conditions. We propose that IRE1α hyperactivation by chronic high glucose results in selective degradation of insulin mRNA, leading to glucose toxicity. It has been shown that insulin mRNA degrades rapidly under ER stress conditions in pancreatic β-cells [16]. However, the precise mechanism whereby IRE1-mediated insulin mRNA degradation occurs is unclear. The reduction of insulin mRNA under ER stress conditions may be initiated by direct endonucleolytic cleavage by the nuclease domain of IRE1, ultimately leading to degradation of the insulin message. Alternatively, IRE1 may function in the activation or recruitment of additional ribonucleases that can degrade insulin messages. It is also possible that IRE1 signaling may somehow initiate insulin gene-specific transcriptional stalling. Regardless of the precise mechanism, our data show that IRE1α, a central component of ER stress signaling, has an essential function in the reduction of insulin mRNA.

Numerous studies have implicated PDX-1 and MafA, two transcription factors that are important for insulin gene transcription, in the defective insulin gene expression in β-cells caused by chronic exposure to supraphysiologic concentrations of glucose [17], [18], [19], [20], [21], [22], [23]. Chronic exposure of β-cells to excess glucose decreases PDX-1 gene expression and MafA protein expression, leading to the suppression of insulin gene expression. Our current results suggest that mRNA degradation is an additional contributor to the reduction in insulin gene expression observed upon chronic exposure to high glucose. All of these effects may act synergistically to decrease insulin mRNA.

Chronically high levels of glucose also cause oxidative stress, leading to activation of c-Jun N-terminal protein kinase (JNK). This JNK activation suppresses PDX-1 binding to the insulin promoter and reduces insulin gene expression [24]. We have shown previously that in mammalian cells ER stress signaling activates JNK through IRE1 [25]. Thus, hyperactivation of IRE1α by chronic high glucose may suppress insulin gene expression partially through JNK-mediated PDX-1 inactivation.

Our work demonstrates that two distinct activities are elicited by high-glucose-induced activation of IRE1α in pancreatic β-cells. IRE1α can be activated in β-cells by overexpressing insulin; and moreover, the level of activation positively correlates with the amount of insulin (Lipson and Urano, unpublished observations). We therefore believe that exposure of β-cells to high glucose levels causes ER stress due to an increased load of insulin translation into the ER. In earlier studies, we found that IRE1α signaling activated by acute exposure to high glucose enhances proinsulin biosynthesis [26]. In contrast, chronic exposure of β-cells to high glucose causes hyperactivation of IRE1α, leading to the degradation of insulin mRNA. Thus, we propose that the duration of exposure to high glucose, and therefore the relative load of translocating insulin, is the critical determinants of the activity of IRE1α.

High glucose exposure causes ER stress and upregulation of ER folding machinery. Insulin mRNA expression increases, but only to a point. This point may represent the time when the burden that the translocating insulin is placing on the ER exceeds the ER processing capacity. In this scenario, the “classical” solution would be to activate Xbp-1 splicing and synthesize more ER folding machinery. However, this upregulation of ER stress-response proteins may add to the burden of the already overloaded ER. The rapid degradation of insulin mRNA could provide immediate relief to the ER and free the translocation machinery. Thus, this mechanism may be an essential element in the adaptation of β-cells to chronic hyperglycemia.

Chronic ER stress has recently been defined as any persistent (on the order of days to years) stress that requires long term adjustments in cellular function [4]. For cells to survive under a chronic ER stress condition like prolonged hyperglycemia, they must have a mechanism by which ER stress can be continuously tolerated. A small number of cells may die, but the majority of cells must survive and adapt to the stressful stimulus, which, in this case, is chronic exposure to high glucose levels. This adaptation may be crucial for the maintenance of β-cell homeostasis and may, in part, explain why the β-cells of Type 2 diabetic patients with chronic hyperglycemia stop producing insulin without simply undergoing apoptosis.

The IRE-mediated mRNA decay pathway may not, however, be limited to a stress- response function. In other types of secretory cells, this mechanism of selective degradation of mRNAs by IRE1 may be effective way to quickly control levels of secretory proteins. In addition to transducing the ER stress response, secretory cells may also activate IRE1 in response to various cellular stimuli, allowing adaptation to rapidly changing physiological conditions. In β-cells, it remains to be seen whether these reductive effects of IRE1α on insulin mRNA are actually important for insulin protein biogenesis, which is regulated at many levels. Modulation of levels of its message is certainly one place for regulation. If the load of insulin folding and processing is exceeding the capacity of the ER, then rapid reduction of insulin mRNA would preserve ER homeostasis and help ensure that the insulin already inside the ER can be properly folded and secreted. This mechanism may also be involved in suppression of the autoimmune response by reducing the amounts of misfolded insulin, which could be a source of “neo-autoantigens.”

Based on recent studies and the results reported here, we suggest that in β-cells IRE1α selectively degrades insulin, the most prevalent ER-targeted mRNA, under adverse conditions. This may be part of a protective adaptation that β-cells have uniquely acquired to protect themselves from death caused by the chronic and high workload placed on the ER under prolonged hyperglycemic conditions. This, combined with subsequent upregulation of ER stress response genes, may function in support of β-cell survival under extreme stress conditions such as chronic hyperglycemia.

## Methods

### Cell culture and transfection

Rat insulinoma cells, INS-1 832/13, and mouse islets were cultured in RPMI 1640 supplemented with 10% FBS. The Cell Line Nucleofector™ Kit T with the Nucleofector Device (Amaxa Biosystems, Gaithersburg, MD) was used to transiently transfect cells. COS7 cells were cultured in DMEM supplemented with 10% FBS and transfected using the FuGene Transfection Reagent (Roche, Basel, Switzerland)

### Immunoblotting

Cells were lysed in ice-cold M-PER buffer (PIERCE, Rockford, IL) containing protease inhibitors for 15 min on ice. The lysates were then cleared by centrifuging the cells at 13,000 g for 15 min at 4°C. Lysates were normalized for total protein (10 µg per lane), separated using 4%–20% linear gradient SDS-PAGE (Bio Rad, Hercules, CA), and electroblotted. Anti-phospho IRE1α antibody was generated from bulk antiserum by affinity purification, followed by adsorption against the nonphospho analog column peptide (Open biosystems, Huntsville, AL). The peptide sequence for generating the antibody was CVGRH (pS) FSRRSG. This phosphopeptide was synthesized, multi-link-conjugated to KLH, and used to immunize 2SPF rabbits. Rabbit anti-total-IRE1α antibody (B9134) was generated using a peptide, EGWIAPEMLSEDCK. Anti-actin antibody was purchased from Sigma (St. Louis, MO).

### Isolating islets from mouse pancreata

Mice were anesthetized by intraperitoneal injection of sodium pentobarbital. Pancreatic islets were then isolated by pancreatic duct injection of 500 U/ml of collagenase solution followed by digestion at 37°C for 40 min with mild shaking. Islets were washed several times with HBSS, separated from acinar cells on a discontinuous Ficoll 400 gradient, viewed under a dissecting microscope, and hand-selected.

### Real-time polymerase chain reaction

Total RNA was isolated from the cells by using RNeasy Mini Kit (Qiagen), then reverse- transcribed using 1 µg of total RNA from cells with Oligo-dT primer. For the thermal cycle reaction, the iQ5 system (BioRad) was used at 95°C for 10 min, 40 cycles at 95°C for 10 sec, and at 55°C for 30 sec. The relative amount of each transcript was calculated by a standard curve of cycle thresholds for serial dilutions of cDNA sample and normalized to the amount of actin. PCR was done in triplicate for each sample. The following sets of primers and Power SYBR Green PCR Master Mix (Applied Biosystems) were used for real-time PCR: for mouse actin, GCAAGTGCTTCTAGGCGGAC and AAGAAAGGGTGTAAAACGCAGC; for mouse insulin1, GAAGTGGAGGACCCACAAGTG and CTGAAGGTCCCCGGGGCT; for mouse insulin2, TGCTGATGCCCTGGCCTGCTCT and CTGGTCCCACATATG CACATGCA; for mouse CHOP, CCACCACACCTGAAAGCAGAA and AGGTGAAAGGCAGGGACTCA; for mouse total XBP-1, TGGCCGGGTCTGCTGAGTCCG and GTCCATGGGAAGATGTTCTGG; for mouse spliced XBP-1, CTGAGTCCGAATCAGGTGCAG (original CAG sequence was mutated to AAT to reduce the background signal from unspliced XBP-1) and GTCCATGGGAAGATGTTCTGG; for rat actin, GCAAATGCTTCTAGGCGGAC and AAGAAAGGGTGTAAAACGCAGC; for rat Glut2, GTGTGAGGATGAGCTGCCTAAA and TTCGAGTTAAGAGGGAGCGC; for rat insulin 1, GTCCTCTGGGAGCCCAAG and ACAGAGCCTCCACCAGG; for rat insulin 2, ATCCTCTGGGAGCCCCGC and AGAGAGCTTCCACCAAG; for rat spliced XBP-1, CTGAGTCCGAATCAGGTGCAG (original CAG sequence was mutated to AAT to reduce the background signal from unspliced XBP-1) and ATCCATGGGAAGATGTTCTGG.

### mRNA Degradation

Cellular mRNA transcription was attenuated by treating cells with 100 µg/mL actinomycin D (Sigma A-4262) for 1 hr followed by treatment with 1 µM thapsigargin for different times. Total RNA was isolated from the cells using the RNeasy Mini Kit (Qiagen). Reverse-transcribed RNA was subjected to real-time PCR quantitation to measure levels of insulin gene transcripts. The relative amount of each transcript was calculated by a standard curve of cycle thresholds for serial dilutions of cDNA sample and normalized to the amount of actin. Time point zero for each condition was standardized to 1 and the subsequent rate of degradation of mRNA was measured.

### Generation of IRE1α heterozygous mice

Mice heterozygous for the *Ire1α* (*Ire1α* ^+/−^) gene [25] were backcrossed into 129SvEv mice more than ten generations to obtain an essentially congenic 129SvEv genetic background.
